# Long-term outcomes following mitral valve replacement in children at heart center Leipzig: a 20-year analysis

**DOI:** 10.1186/s13019-024-02904-7

**Published:** 2024-07-03

**Authors:** Katja Schumacher, Mateo Marin-Cuartas, Muhammed Ikbal Aydin, Manuela de la Cuesta, Sabine Meier, Michael Andrew Borger, Ingo Dähnert, Martin Kostelka, Marcel Vollroth

**Affiliations:** 1 Department of Cardiac Surgery, Leipzig Heart Center, Leipzig University, Leipzig, Germany; 2 Department of Pediatric Cardiology, Leipzig Heart Center, Leipzig, Germany

**Keywords:** Pediatric cardiac surgery, Mitral valve replacement, Mitral valve disease, mitral valve surgery

## Abstract

**Background:**

Although mitral valve repair is the preferred surgical strategy in children with mitral valve disease, there are cases of irreparable severe dysplastic valves that require mitral valve replacement. The aim of this study is to analyze long-term outcomes following mitral valve replacement in children in a tertiary referral center.

**Methods:**

A total of 41 consecutive patients underwent mitral valve replacement between February 2001 and February 2021. The study data was prospectively collected and retrospectively analyzed. Primary outcomes were in-hospital mortality, long-term survival, and long-term freedom from reoperation.

**Results:**

Median age at operation was 23 months (IQR 5–93), median weight was 11.3 kg (IQR 4.8–19.4 kg). One (2.4%) patient died within the first 30 postoperative days. In-hospital mortality was 4.9%. Four (9.8%) patients required re-exploration for bleeding, and 2 (4.9%) patients needed extracorporeal life support. Median follow-up was 11 years (IQR 11 months − 16 years). Long-term freedom from re-operation after 1, 5, 10 and 15 years was 97.1%, 93.7%, 61.8% and 42.5%, respectively. Long-term survival after 1, 5, 10 and 15 years was 89.9%, 87%, 87% and 80.8%, respectively.

**Conclusion:**

If MV repair is not feasible, MV replacement offers a good surgical alternative for pediatric patients with MV disease. It provides good early- and long-term outcomes.

## Introduction

In children with mitral valve (MV) disease reconstructive techniques are the therapy of choice and in most cases achievable with good outcomes. Nevertheless, there are cases where repair is not feasible due to severely dysplastic valves [1]. In these cases, valve replacement is the only remaining option although the ideal valve prosthesis for children is yet to be found. Mechanical MV replacement is most commonly performed since these are offered in smaller sizes and are also more durable than bioprostheses [2]. Especially in small children, MV replacement is technically demanding and will eventually result in the need for re-replacement due to the patient’s growth [1, 2]. Besides the need for redo surgery, in older publications MV replacement has been associated with high mortality rates ranging from 10 to 36% [1–3].

The aim of this study is to analyze the early postoperative outcomes as well as long-term survival and freedom from reoperation following mitral valve replacement in children during a time period of 20 years at the Leipzig Heart Center.

## Patients and methods

### Ethical statement

This research project was approved by the ethics committee from the University of Leipzig in accordance with the Declaration of Helsinki (protocol number 120/23-ek). Individual patient informed consent was waived due to the anonymous data management and the retrospective nature of this study.

### Study cohort

Pediatric patients undergoing MV replacement between February 2001 and February 2021 were included in our analysis. Data were prospectively collected in the institutional database and retrospectively analyzed.

### Surgical technique

All operations were performed through median sternotomy. Surgery was performed on cardiopulmonary bypass with aortic and bicaval cannulation in moderate hypothermia (rectal temperature 28 °C). Cardioplegic arrest was achieved using St. Thomas Hospital cardioplegia solution (35 ml/kg body weight, repeated application of 25 ml/kg body weight after 90 min cardioplegic arrest). A vent was placed in the right upper pulmonary vein. For optimal MV visualization, right atrial transseptal approach was used in all patients. All mitral valve components, including leaflets, chords and papillary muscles were excised. Interrupted pledgeted sutures were placed into the native mitral valve annulus or supraannular in patients with very low annular diameter. In all patients, the prosthetic valves were placed in an anti-anatomic fashion. The leaflets were carefully tested before closing the interatrial septum and the right atrium. After weaning from cardiopulmonary bypass, valve function and transvalvular gradients were evaluated with transesophageal echocardiography.

### Follow-up

Right after mitral valve replacement, sufficient anticoagulation was achieved using unfractionated heparin (target PTT 60–80). After that, for mechanical prostheses life-long anticoagulation was recommended with vitamin K antagonists aiming for a target INR of 2.5–3.5. Until the target INR is reached, overlapping heparin administration was mandatory. In case of biological prostheses, anticoagulation was recommended for three months postoperatively.

Patients’ demographics, baseline characteristics, and surgical data were obtained from our institutional medical records. Follow-up data were collected during subsequent visits in our outpatient clinic or by the referring pediatric cardiologists.

### Statistical methods

Categorical variables are expressed as frequencies and percentages throughout the manuscript. Continuous variables are expressed as mean ± standard deviation for normally distributed variables and median and interquartile range (IQR) for non-normally distributed variables. Statistical analyses were performed using Microsoft Excel 2019. Freedom from reoperation and survival rate was estimated using the Kaplan–Meier method. Statistical analyses were performed using the SPSS software package, version 25.0 (IBM Corp, Armonk, NY, USA) and Microsoft Excel 2019 for Mac.

## Results

### Patient baseline characteristics

A total of 41 consecutive pediatric patients undergoing MV replacement the study period were included in the analysis. Baseline characteristics are shown in Table 1. The median age at operation was 23 months (IQR 5–93 months) and the median weight was 11.3 kg (IQR 4.8–19.4 kg). A total of 7 (17.1%) patients were younger than 3 months. Patent ductus arteriosus (*n* = 13, 31.7%) and atrioventricular septal defect (*n* = 11, 26.8%) were the most common concomitant congenital heart defects. A total of 33 (78.6%) patients underwent previous cardiac surgery prior to mitral valve replacement. Beside previous MV repair either isolated or in more complex operations, the most common surgeries performed prior to MV replacement were for correction of aortic coarctation (*n* = 9, 22%) and atrioventricular septal defect (*n* = 11, 26.8%). One patient (2.4%) with a complex transposition of the great arteries received pulmonary banding prior to arterial switch operation. Indication for MV replacement was infective endocarditis in 5 patients (12.2%) from which 4 patients showed native valve endocarditis (9.8%). In our population, 12 patients (29.3) had severely dysplastic valves resulting in hemodynamic relevant MV regurgitation in 8 (19.5%) and MV stenosis in 4 patients (9.8%). Prosthesis stenosis after prior MV replacement was indication for surgery in 4 patients (9.8%). In 20 (48.8%) cases MV repair prior to the index surgery did not achieve satisfactory long-term results due to significant MV regurgitation.

**Table 1:** Baseline characteristics

Age (months)	23 (5–93)	
Age category		
0–30 days	3 (7.3)	
1–3 months	4 (9.8)	
4–6 months	4 (9.8)	
7–12 months	2 (4.8)	
1–2 years	8 (19.5)	
2–6 years	6 (14.6%)	
7–10 years	7 (17.1)	
11–18 years	7 (17.1)	
Weight - kg	11.3 (4.8–19.4)	
Female	27 (65.9)	
Previous pulmonary artery banding	1 (2.4)	
Previous cardiac surgery	33 (78.6)	
Shone complex	6 (14.6)	
Down syndrome	2 (4.8)	
AVSD	11 (26.8)	
Atrial septal defect type II	9 (22)	
Patent ductus arteriosus	13 (31.7)	
Patent foramen ovale	10 (24.4)	
Aortic anomalies	9 (22)	

Categorical variables are presented as frequencies and percentages in parentheses. Continuous variables are presented as median and interquartile range in parentheses. **AVSD**: atrioventricular septal defect; **IQR**: interquartile range

### Intraoperative variables

Intraoperative details are presented in Table 2. The median cardiopulmonary bypass time was 132 min (IQR 109–152) and the median aortic cross-clamp time was 73 min (IQR 62–89). Whereas most patients underwent MV replacement as initial strategy (*n* = 38, 92.7%), three patients (7.3%) underwent replacement due to failed MV repair. Only one patient (*n* = 1; 2.4%) received a bioprosthetic MV replacement. Median size of the valve was 18 mm (IQR 16–25), median valve size/weight ratio was 1.8 (IQR 1.1–3.4).

**Table 2:** Intraoperative details

CPB time - minutes	132 (109–152)
Aortic cross-clamp time - minutes	73 (62–89)
Temperature (Celsius)	28 (26–28)
Mechanical valve – n (%)	40 (97.6)
Type of the valve	
Medtronic ATS	28 (68.3)
Medtronic Mosaic	1 (2.4)
Abbott SJM	12 (29.3)
Valve size - mm	18 (16–25)
12	1 (2.4)
15	5 (12.1)
16	12 (29.3)
17	2 (4.9)
18	2 (4.9)
19	2 (4.9)
20	1 (2.4)
23	3 (7.3)
25	9 (22.0)
27	2 (4.9)
29	1 (2.4)
31	1 (2.4)
Size/weight ratio	1.8 (1.1–3.4)
Secondary sternal closure – n (%)	9 (22.0)

Categorical variables are presented as frequencies and percentages in parentheses. Continuous variables are presented as median and interquartile range in parentheses. **CPB**: cardiopulmonary bypass time; **IQR**: interquartile range

### Early postoperative outcomes

One (2.4%) patient died within the first 30 postoperative days. The in-hospital mortality was 4.9%. The median intensive care unit (ICU) stay was 9 days (IQR 6–17). A total of 4 (9.8%) patients presented with major postoperative bleeding requiring surgical re-exploration and 2 (4.9%) patients required post-operative mechanical circulatory support with veno-arterial extracorporeal membrane oxygenation due to postcardiotomy cardiogenic shock. Permanent pacemaker implantation was performed in 7 (17%) patients. Four (9.8%) patients developed chylothorax postoperatively. Further early postoperative outcomes are summarized in Table 3.

**Table 3:** Postoperative outcomes

Postoperative complications	
Chylothorax	4 (9.8)
Pacemaker	7 (17)
ECMO	2 (4.9)
Bleeding requiring re-exploration	4 (9.8)
**Postoperative outcomes**	
In-hospital mortality - %	4.9
30-day mortality - %	2.4
ICU stay - days	9 (6–17)
Hospital stay - days	24 (17–34)
**Indications for redo MV replacement during follow-up**	
MV stenosis	10 (24.4)
MV regurgitation	1 (2.4)

Categorical variables are presented as frequencies and percentages in parentheses. Continuous variables are presented as median and interquartile range in parentheses. **ECMO**: extracorporeal membrane oxygenation; **ICU**: intensive care unit; **IQR**: interquartile range

### Follow-up

**Fig. 1:**
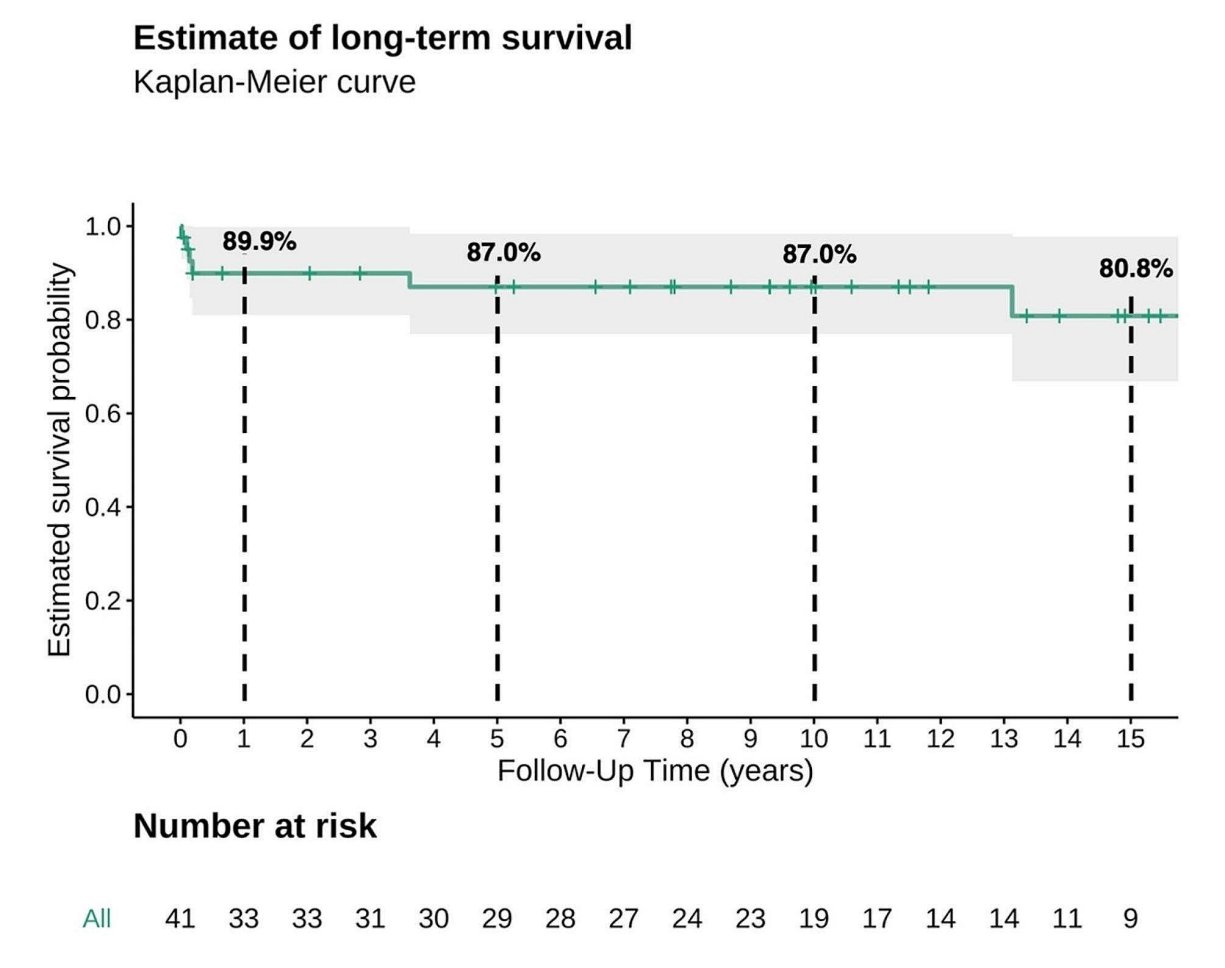
Kaplan meier estimate of long-term survival (%)

**Fig. 2:**
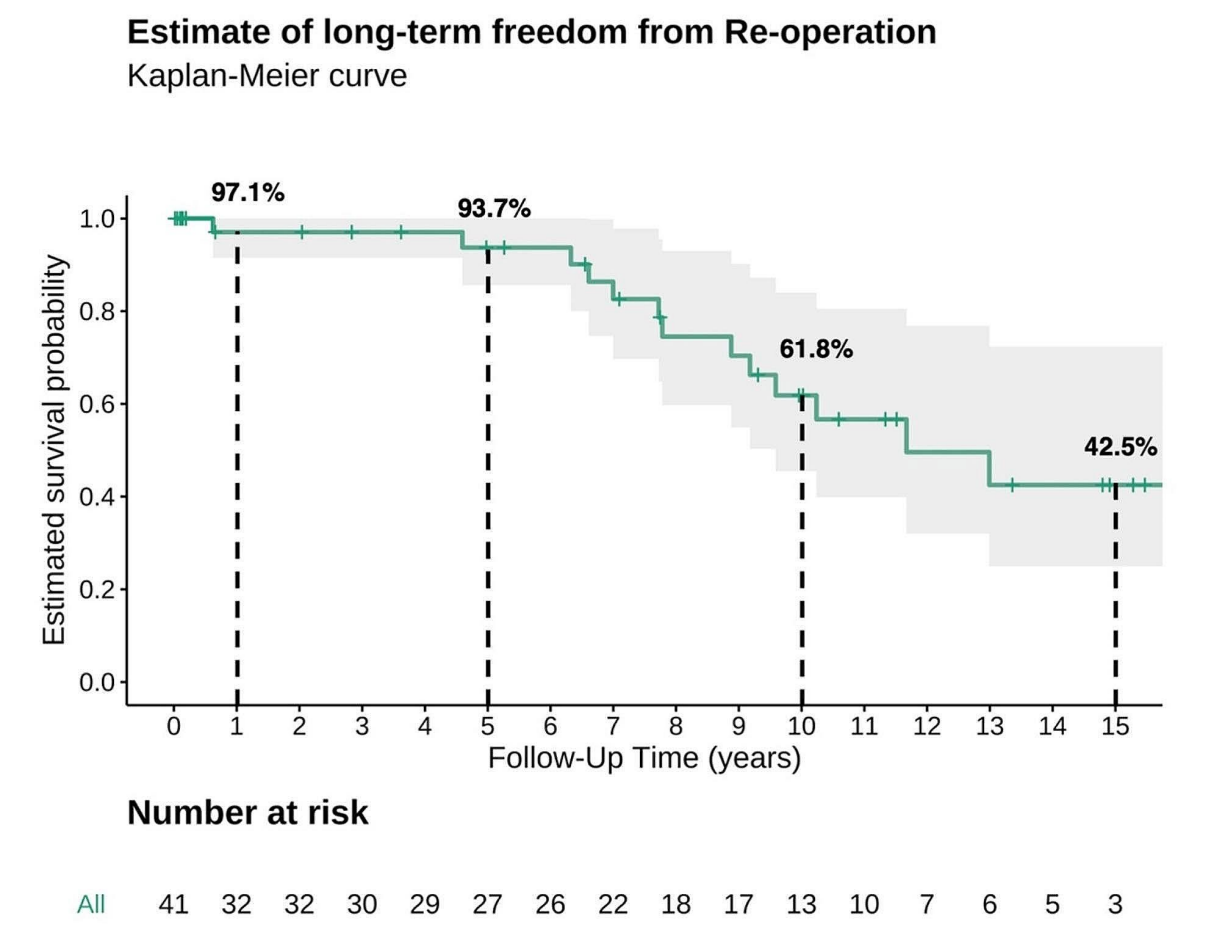
Kaplan meier estimate of the long-term freedom from re-operation (%)

The median follow-up was 11 years (IQR 11 months − 16 years). Estimated long-term survival at 1, 5, 10, and 15 years was 89.9%, 87%, 87% and 80.8%, respectively (Fig. 1). Overall six patients (14.6%) died during follow-up (Table 4). Estimated freedom from re-operation at 1, 5, 10 and 15 years was 97.1%, 93.7%, 61,8% and 42.5%, respectively (Fig. 2). The main cause of reoperation was prosthesis stenosis in 10 cases (24.4%). The one case requiring redo MVR due to mitral regurgitation was the only patient that received a biological prosthesis during the initial surgery. The median time to reoperation was 95 months (IQR 80–117 months).

**Table 4:** Causes of death

Intracerebral hemorrhage	2 (4.9%)
Gastrointestinal hemorrhage	1 (2.4%)
Sepsis	1 (2.4%)
Unknown	2 (4.9%)

## Discussion

The current study represents a single-center experience over a period of 20 years in pediatric patients undergoing MV replacement. The main findings of our study are:

1. The most frequent cause of reoperation is MV stenosis (i.e., patients’ outgrowth the MV prosthesis).
2. 61.8% of the patients are free from reoperation after 10 years.
3. Nevertheless, long-term survival is not strongly affected by the redo procedures.

MV diseases needing surgical interventions are rare in children, but sometimes MV replacement becomes inevitable after failed repair or in cases of severe valve dysplasia. Despite advances in surgical techniques as well as valve prostheses, MV replacement in children is still associated with high mortality rating from 10 to 36% in the literature [2, 3]. In-hospital mortality in our cohort was with 4.9% (*n* = 2) lower than described in most studies. Both children were less than a week old at the point of surgery and died from intracerebral hemorrhage. The variability of mortality rated in different studies could be explained with the age of the included patients. *Brancaccio et al.* [4]. described an association between increased mortality and age < 2 years. In our cohort, 21 children (51.2%) were younger than 2 years and all the children who died within the first 90 days after MV replacement belonged to that age group supporting this association [5]. However, we did not prove statistical significance due to the low event rate. Another described risk factor for early post-operative mortality is the size/weight ratio of the implanted valve > 2 [4]. These findings also align with the results in our cohort since the two patients who died early after surgery weighed less than 5 kg and received mechanical valves with a size of 15–16 mm (valve size/weight ratio > 5). Corresponding with other studies, most deaths after MV replacement in our cohort occurred early after surgery [1, 4]. Given the fact that a significant number of patients require redo surgery, long-term survival after 10 years was acceptable with 87% in our cohort. This finding goes along with other studies which reported survival rates around 75% [1, 4, 6]. Again, valve size/weight ratio is described as a risk factor for decreased survival. Ibarra et al. [2] showed that patients with size/weight ratio 1 to 2 had longer reoperation-free survival than patients with a ratio less than 1 or greater than 2 (*P* < .001). In their study, reoperation-free survival at 5 years was 96% for patients with a ratio from 1 to 2 and 46% for patients with a ratio less than 1 or greater than 2. In our cohort, most patients who deceased during follow-up had a size/weight ratio far over 2, although we cannot prove statistical significance due to the small event rate. Freedom of re-operation rates after 10 years from 50 to 72% in the literature [1, 4, 6] which also align with our own experience in the presented cohort.

To this point, there are still only a few prosthetic valves available for young children with small MV annuli and the ideal prosthesis is yet to be found. On the one hand, mechanical prosthetic valves provide good durability yet on the expense of complications due to long-term anticoagulation [7]. Furthermore, patients with non-dysfunctional mechanical valves might also require redo MV replacement because of somatic growth especially if the initial MV replacement is performed with small valves due to low weight. Valve oversizing is often performed intending to delay redo surgery. However, there is data suggesting that the use of a larger prostheses in patients with small mitral annulus is associated with several negative outcomes such as increased risk of perioperative mortality as well as complications because of left ventricular outflow tract obstruction, prosthetic leaflet entrapment with increased risk of prosthesis thrombosis, circumflex artery injury, and damage to the conduction system [2, 4]. On the other hand, bioprosthetic valves are attractive as they require no permanent anticoagulation. However, they do degenerate faster in young children with a median time to redo replacement ranging from 2.5 to 5 years [8]. The most common reason of structural valve degeneration in both porcine and bovine pericardial bioprosthetic valves is mitral stenosis due to calcification and pannus formation [8, 9]. Furthermore, classic bioprosthetic valves present the same size issue as mechanical valves, especially since some models have even higher profiles leading to problems such as left ventricular outflow tract obstruction [8]. Particularly in very young children with annular hypoplasia, bovine jugular vein conduits (Melody valve; Medtronic) could represent an eligible alternative with good early outcomes as well as the possibility of subsequent valve expansion delaying redo MV replacement [9, 10]. This procedure could be used as a bridge therapy to allow growth of the mitral annulus and following fitting of a size appropriate mechanical prosthesis later in life [4].

## Limitations

There are several limitations of this study that should be noted. First, this is a single‑center retrospective study with the corresponding limitations associated with its nature. Moreover, the study does not compare different treatment strategies Additionally, our study reports the results from a high-volume center with a wide experience treating heart valve disease in pediatric populations. Hence, our results might not be generalizable to smaller centers with lower operative volumes. Since there is a lack of comparable echocardiographic follow-up, the development of transvalvular gradients and structural heart changes over time during the growing phase of the pediatric population cannot be evaluated. Finally, given the long-term retrospective nature of the study and, therefore, markedly differing follow-up periods among patients and a limited cohort size, more complex statistical analyses were not feasible.

## Conclusion

If MV repair is not feasible, MV replacement offers a good surgical alternative for pediatric patients with MV disease. It provides good early- and long-term outcomes. Although several patients required MV reoperation due to patients’ growth, long-term survival is not strongly affected by the redo procedures.

## Data Availability

The data that support the findings of this study are not openly available due to reasons of sensitivity and are available from the corresponding author upon reasonable request.

## Abbreviations

AVSD: Atrioventricular septal defect
CBP: Cardiopulmonary bypass
ECMO: Extracorporeal membrane oxygenation
ICU: Intensive care unit
IQR: Interquartile range
MV: Mitral valve
